# A Kinematic Information Acquisition Model That Uses Digital Signals from an Inertial and Magnetic Motion Capture System

**DOI:** 10.3390/s22134898

**Published:** 2022-06-29

**Authors:** Andrea Catherine Alarcón-Aldana, Mauro Callejas-Cuervo, Teodiano Bastos-Filho, Antônio Padilha Lanari Bó

**Affiliations:** 1 Ph.D. Program in Engineering, Universidad Pedagógica y Tecnológica de Colombia, Tunja 150002, Colombia; 2 Faculty of Engineering, Universidad Pedagógica y Tecnológica de Colombia, Tunja 150002, Colombia; mauro.callejas@uptc.edu.co; 3 Postgraduate Program in Electrical Engineering, Federal University of Espírito Santo, Vitória 29075-910, Brazil; teodiano.bastos@ufes.br; 4 School of Information Technology and Electrical Engineering, The University of Queensland, Brisbane 4072, Australia; antonio.plb@uq.edu.au

**Keywords:** motion capture, inertial magnetic sensors, signal processing, kinematics, articular amplitude, upper limb, optical motion capture, optical analysis

## Abstract

This paper presents a model that enables the transformation of digital signals generated by an inertial and magnetic motion capture system into kinematic information. First, the operation and data generated by the used inertial and magnetic system are described. Subsequently, the five stages of the proposed model are described, concluding with its implementation in a virtual environment to display the kinematic information. Finally, the applied tests are presented to evaluate the performance of the model through the execution of four exercises on the upper limb: flexion and extension of the elbow, and pronation and supination of the forearm. The results show a mean squared error of 3.82° in elbow flexion-extension movements and 3.46° in forearm pronation-supination movements. The results were obtained by comparing the inertial and magnetic system versus an optical motion capture system, allowing for the identification of the usability and functionality of the proposed model.

## 1. Introduction

Motion capture systems are widely used in different fields and disciplines, including film, animation, video games, health (mainly in rehabilitation processes), and sports science, among others [1,2,3]. These systems have evolved from the appearance of the rotoscope in 1915, used in the entertainment industry [4], to the latest systems with increasingly better characteristics used in various knowledge fields. There are electromechanical, electromagnetic, inertial, and optical motion capture systems with markers (active and passive), and without markers. Each of these systems has unique characteristics and advantages in the acquisition of motion-related signals [5,6,7] and consists mainly of specialized hardware and software [8].

Three-dimensional motion capture systems or optoelectronic systems, such as Vicon (Oxford, UK), MotionAnalysis (Santa Rosa, CA, USA), OptiTrack (Corvallis, OR, USA), or Qualisys (Göteborg, Sweden) [9], offer precision and reliability, among other advantages. As a result, optoelectronic systems have become a gold standard and have been used as benchmark systems in the validation of other proposals, especially in fields related to human healthcare [10,11,12,13,14,15].

Among the criticized features of optoelectronic systems are their high cost, setup complexity, occlusion problems, and the space requirement in a controlled environment for data acquisition, making them unsuitable for use in ambulatory environments or large capture areas [16]. Due to the advances in sensing and other fields that are enriching and changing some aspects of motion science, new alternatives for motion sensing have emerged, including inertial and magnetic systems that offer portability, autonomy, and affordability [17,18]. Nowadays, the contribution of inertial systems in this field is widely recognized. However, in the motor rehabilitation field and beyond, motion capture is necessary to estimate the angle between human joints and the rotations associated with body movements, which provides useful information for kinematic analysis and functional performance quantification.

Some related research below focuses on functional performance quantification. For example, a calibration protocol for capturing the kinematic data of the thorax and upper limbs in children is described in [19]. This protocol is oriented to the development of a coordinate system for motion analysis. In addition, using a Levenberg-Marquardt algorithm, a function for obtaining the corresponding rotation matrices is presented. Furthermore, to provide higher accuracy and mitigate the negative impact of external accelerations and ferromagnetic disturbances that disturb systems based on inertial sensors, several filters are applied, prevailing the Kalman-based filters.

Specifically, in [20], a real-time three-dimensional human motion reconstruction method is presented. The orientation of the arm and forearm is obtained from the proposed orientation method, which is implemented in Python and includes two parallel adaptive Kalman filters with perturbation models. The method adjusts the noise covariance matrix, performs a verification process to discard outliers, and finally calculates the orientation quaternion by means of a triaxial attitude determination algorithm.

Similarly, for the case of the lower limbs, a method for estimating movements is presented in [21], particularly for the gait analysis in a controlled environment. The method is based on a previously proposed approach [22], in which the human hip angles are estimated relative to a vertical reference mark. A portable motion capture device was built in [23], integrating two flow microsensors, an accelerometer, and a gyroscope to determine the lower-limb motion rate, acceleration, and attitude angle (elevation angle). With the use of a single device on the lower limb, and applying a neural network model for intra-limb coordination, the thigh movement is also determined in real-time.

In other works similar to [24], and using only two inertial sensors (one located on the leg and the other on the back), it is possible to obtain data and model the kinematics from standing to sitting movements, classifying these transitions. Based on the approaches outlined in [25], a tool is provided to track the health status of people in a residential environment in the UK, describing a method comparable to the extended Kalman filter (EKF), constrained EKF, and ERPF without transition constraints. This allows for the estimation of human limb angle, angular velocity, acceleration, and associated standard deviations using accelerometers across the second segment of a two-link chain (e.g., forearm or leg). In this way, from a parametric model to obtain the kinematics of the human body and using the kinematic variables, a pattern of activities can be established to help detect abnormal situations and promptly take corrective measures.

Currently, there is an increasing focus on estimating joint angles with inertial measurement units (IMUs). For this reason, 112 studies published between 2000 and 2018 were systematically reviewed and categorized according to the methods used to derive the orientation of bony anatomy when estimating joint angles [26]. Assumed alignment methods were used in 42% of the papers, while functional alignment methods were the most commonly used (47% of the papers). Likewise, 7% of the papers used model-based methods, while only 4% of papers relied on augmented data methods, indicating that there is still a need to create and adopt standards for defining anatomical axes to improve the accuracy of biomechanical information. With a method that allows the digital signals generated by the inertial and magnetic motion capture system to be converted and transformed into kinematic information, the interpretation and analysis of the information by health and sports professionals can be performed more easily and rapidly without the need for additional procedures or scientific knowledge to post-process the data.

For this reason, starting from the fact that inertial systems provide information in technical language, this article proposes a method that allows the signals generated by an inertial-magnetic motion capture system to be converted into kinematic information (expressed in degrees corresponding to the specific movements of the body segment) that can be easily interpreted by professionals in the field of physical rehabilitation. To achieve this, the authors use a system named Imocap-GIS [27,28,29], which has been used in several projects and has made a significant contribution to the use of inertial-magnetic motion capture systems [30,31,32,33,34]. The use of this technology allows the use of a method to obtain kinematic information directly, reducing the required time, and lessening the data analysis and interpretation processes since specialized personnel and expertise are not always required.

The proposed transformation method consists of five stages after the capture of the basic signals obtained by the sensors (accelerations, angular velocities, and magnetic field velocities): (i) the process begins with the data transmission process from the Imocap-GIS motion capture system; (ii) message routing is performed in the external device receiving the data (e.g., a computer); (iii) a method of kinematic representation of orientation and position of an object is selected (quaternions and/or Euler angles); (iv) the rotations are assigned according to the analytical units of the upper-limb movement (elbow-joint flexion, extension, pronation, and supination); and, finally, (v) the structure for the visualization of the biomechanical information in a virtual environment is established. As part of the validation process of the proposed method, a test protocol is applied to determine the root mean square error (RMSE) in obtaining the joint amplitude and measurement of elbow movements, using the inertial and magnetic system versus the data obtained with an optical motion capture system.

## 2. Materials and Methods

A motion capture system, through the application of a set of techniques, allows the acquisition of data related to the movement of a subject (human, animal, or other) and the reproduction of these data in a computational environment, providing specific information that requires processing by professionals in the technical field. The main material used in this research was the Imocap-GIS [27] motion capture system described in this section and the proposed method for converting the digital signals generated by Imocap-GIS.

### 2.1. Description of the Imocap-GIS Inertial and Magnetic System

**Figure 1:**
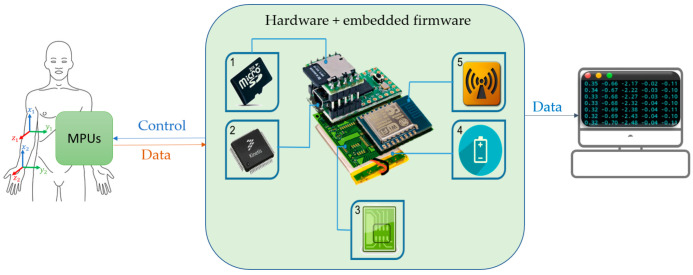
Hardware components of the Imocap-GIS device. 1. Mass Storage; 2. Main Processor (SoC); 3. Printed Circuit Board; 4. Power Source and Management; 5. Modem and Radio Modules.

Imocap-GIS is a signal acquisition and processing system that includes motion processing units (MPUs) that generate digital signals. Operations, such as data fusion, transformations to represent the direction of motion (quaternions, Euler angles, etc.), and signal processing using the Kalman filter are performed on these signals. In response, the system provides numerical values that are sent to an external device (e.g., a PC) for further analysis, as shown in Figure 1. Table 1 contains a general description of the main components of the motion capture system. It is worth noting that the values obtained with the Imocap-GIS system have been validated in previous work using reference systems, such as an ABB IRB 120 industrial robot arm, the Biometrics SG110 commercial electrogoniometer, and the Qualisys optoelectronic system [27,35,36], experiments that provided reliable results for the use of this system.

The motion capture system stores the data generated by the sensors on an external storage card (micro SD) or sends them directly to the external device in real-time. In any case, for each sensor connected to the system, a plain text file is generated with three data columns (if the data are generated in Euler angles), or four columns (if they are generated using quaternions). The data can be analyzed and used in different contexts, but require prior arrangements and processing to achieve an understandable format for the end-users.

### 2.2. Signal Transformation Model

**Figure 2:**
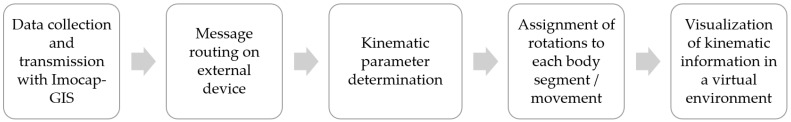
Digital signal transformation method, divided into five stages.

The method for converting digital signals into kinematic information that can be understood by end-users (e.g., physical medicine and rehabilitation professionals) involves five steps (Figure 2). First, the data used by Imocap-GIS are collected, filtered, and transmitted. Then, the routing of the messages occurs in the device that receives the data to subsequently determine the kinematic parameters of the biomechanical model. The rotations are assigned according to the analytical units of the motion of the body segments to be analyzed (in this case, the upper limb, limited to the elbow joint). Finally, the method of visualization of the biomechanical information is performed in a virtual environment.

#### 2.2.1. Stage 1. Data Collection and Transmission Using the Imocap-GIS Motion Capture System

In the initial phase of the motion capture using the Imocap-GIS device, two basic moments are considered: the process of data acquisition and filtering, and the process of data transmission to an external device.

- Data collection and filtering

To initiate data collection, direct, wired, internal, and low-level communication is used between the sensors (MPUs) and the controller. In this process, the sensors send the most recent data corresponding to the current values of the accelerometer, gyroscope, and magnetometer, one packet at a time. The transmission is performed via I2C, a serial digital chip-to-chip communication protocol that allows defining physical connections for bit transfer between integrated circuits without data loss and with a high speed and throughput rate (60–120 Hz) [37].

Once the central unit has the nine most recent data from the three MPU sensors in the three axes (X, Y, Z), the filtering process and signal processing are performed, which changes the inherent characteristics of the data according to the programming or configuration in the firmware of the system (Quaternions/Euler). To minimize errors caused by data drift and fluctuations, filters have been built into Imocap-GIS (mainly Kalman filters, Madwik filters, and hardware filtering using the EM7180), and the filter settings of each sensor are also applied to remove as much noise as possible from the signals.

- Data transmission from Imocap-GIS to an external device

When the above process is complete, the information can be sent to an external device (computer, tablet, mobile phone, etc.). There are both wired (e.g., USB, optic fiber) and wireless (WiFi, Bluetooth, Zigbee, or other communication protocol) alternatives, both based on serial interfaces using the Universal Asynchronous Receiver Transmitter (UART) protocol [38,39]. In the context of this research, wireless alternatives were used for data acquisition, particularly WiFi (IEEE 802.11b/g/n), which shares the same ISM (industrial, scientific, and medical) frequency bands in the 2.4 GHz range with the Bluetooth and Zigbee protocols [40]. The WiFi protocol offers advantages in terms of power consumption, transmission range, cost, ease of use, scalability, and interoperability [41,42], as well as the approval of this protocol in medical environments.

In the Imocap-GIS system, data are sent and received through the TCP/IP model [43], which can operate through two communication protocols in its transport layer: TCP and UDP. Considering the characteristics of these protocols, although TCP has advantages in packet management and tracking [44], the UDP protocol allows direct packet transmission reducing the TCP-related latency [45]. Moreover, it enables the implementation of the broadcast transmission method, where the data are delivered to all the devices (receivers) connected to the same network, which corresponds to the behavior of the Imocap-GIS system.

When the data are received at the destination device, the process involves collecting the packet, decoding, and retrieving the structured information. This process occurs as a result of the data being serialized using the FIFO (first-in, first-out) method, which allows high-speed, low-power data transfers [46].

#### 2.2.2. Stage 2. Message Routing on External Device

Message routing refers to the process of delivering information to the receiver and then addressing it for appropriate use in the required context. After receiving the sensor data, the first step is to define a communication channel, a process that Imocap-GIS can perform in two operating modes, as follows:

- Access point (AP) mode: this mode is enabled when Imocap-GIS does not detect a network. In this case, the system behaves as an access point, creating a new WiFi network on the least busy channel found within the 2.4 GHz frequency.
- Station (STA) mode: in this mode, Imocap-GIS works as a station, i.e., the system is connected to an existing access point (a router, for example), where the transmission frequency is given by the already established WiFi network.

**Figure 3:**
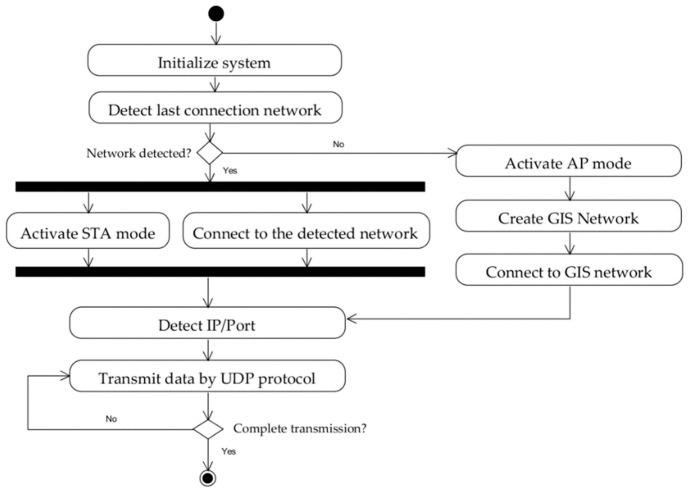
Imocap-GIS operating modes for message routing.

Regardless of the operating mode of the system (STA or AP), message forwarding is handled via IP, i.e., it considers where the data come from and where they go, using the IP addresses of each connected device (server, sensors, etc.). Figure 3 shows the sequence that defines the operating mode of Imocap-GIS.

By default, once Imocap-GIS is activated, it starts searching for a known network and tries to connect to it as specified in the firmware. If found, it will connect to that access point, so the Imocap-GIS system would be in STA mode. Otherwise, AP mode is enabled, and a new WiFi network named “IMOCAP_ESP_ADM” appears after a few seconds. Regardless of the selected operating mode, the system connects to the network, detects the first IP that has been connected, and starts transmitting data to this device via the established port. This process continues cyclically until all sensors that are to be used for data collection are linked. This ensures that the data acquired by the Imocap-GIS system are always sent to the target device via the network.

#### 2.2.3. Stage 3. Kinematic Parameter Determination

The information received from the motion capture system is the raw material for the motion description and its corresponding simulation in a 3D virtual environment, which is achieved from the representation of the orientation and position of a rigid body. A rigid body is a system of many particles that keep a constant distance while the body is in motion following a trajectory, where its position changes with a fixed point reference, also known as the absolute coordinate system [47,48]. The position of the body is determined with the aid of the ***P*** position vector (consisting of two coordinates in the two-dimensional plane or three coordinates in a three-dimensional plane), representing the position of a certain point on the object compared to the zero position of the reference system.

To determine and represent the orientation of the rigid body, three moments are considered: (i) having an absolute reference system (e.g., inertial); (ii) assigning a fixed or local reference system on the rigid body (which moves according to the movements of the rigid body); and (iii) linking the absolute and local reference systems to obtain the information of the orientation of the body at a given time instant.

In this sense, the rotation matrix [49] can be used to relate the reference frames and obtain the orientation representations. It is also possible to use some parameterization methods, either by minimal representations (which include a set of three parameters to describe the orientation), such as the axis-angle representations, by roll, pitch and yaw angles, or by Euler angles [50]. As another alternative, implicit representations can be used (which include constraints in addition to the three minimum parameters), as in the case of the quaternion representation, which uses four parameters [51,52]. In essence, all of these representations lead to the integration of mathematical equations that provide an estimate of the orientation of various objects in a three-dimensional space [53].

In this research, these representations allow for fusing the signals obtained from the triaxial sensors (gyroscope, accelerometer, and magnetometer) integrated with Imocap-GIS in order to translate these data into a representation of the orientation of the body during its movement in 3D space. This process is composed of three steps: (i) system configuration; (ii) determination of the initial position and orientation and reference parameters; and (iii) estimation of the position and orientation when the movement starts.

- Step 1: Initial system setup

**Figure 4:**
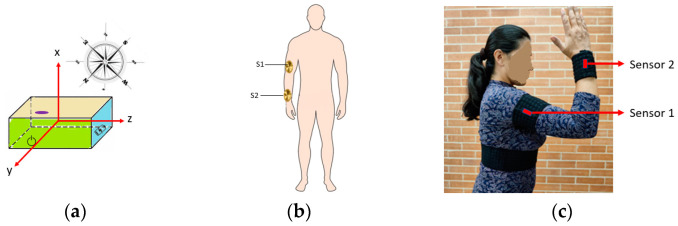
Orientation and location of the Imocap-GIS sensors: (**a**) initial location of the sensor relative to the fixed coordinate system; (**b**) location of the sensors on the body segments in the initial position; (**c**) use of hook-and-loop tape for attaching the sensors to the body segments.

First, each Imocap-GIS unit is positioned in relation to a global coordinate system (determined by magnetic north) based on the magnetometer data, as shown in Figure 4a. This is used to determine the rotation required for each sensor and establish its initial position on the body segments according to the calibrated body posture. The initial position is given by the vertical orientation of the body segments, with the upper extremities at the sides of the body and the hands facing the body. In addition, the pose must be achieved with the head straight and the feet pointing forward (allowing for an upright or seated position), as shown in Figure 4b. This pose was chosen because the joint angles try to reach a position of 0° and the initial calibration can be performed with the highest reliability.

As a case study in this work, the upper limb of the human body was chosen. In this sense, to capture and measure the movements of the elbow and radioulnar joint, two sensors were used, one located on the outer side in the lower third of the arm segment and the other in the lower third of the forearm, supported on the ulna and radius. Both were held in place with an elastic hook-and-loop band, as seen in Figure 4c. The axes of the sensors were initially aligned with the global anatomical reference system, which was composed of three axes: X (with vertical orientation, pointing in the same direction as the Earth’s gravitational force), Y (also called anteroposterior axis, which points to the front), and the Z (or mediolateral, pointing to the right side).

- Step 2: Determination of initial orientation and reference parameters

Before the motion capture, the estimation of the initial orientation was carried out through the measurements of the sensors of the inertial and magnetic unit, from the acceleration measurements (vector denoted as $\vec{a}$*i*(*k*)), angular velocities ($\vec{\omega }$*i*(*k*)), and magnetic fields ($\vec{b}$*i*(*k*)), where *i* is the sensor number (1 for the arm and 2 for the forearm) and *k* is the sample number.

Subsequently, the measurements of each sensor in the anatomical reference frame were obtained using the procedure presented below, which is described in detail in [35,54]. Using a fixed matrix (***R****i*), the orientations of the local and anatomical reference system are related to obtaining the measurements of each sensor in the anatomical reference frame for accelerations (Equation (1)), angular velocities (Equation (2)), and magnetic fields (Equation (3)).

(1) $${\vec{a}}_{ri}\left(k\right)=R_{i}{\vec{a}}_{i}\left(k\right),$$

(2) $${\vec{\omega }}_{ri}\left(k\right)=R_{i}{\vec{\omega }}_{i}\left(k\right),$$

(3) $${\vec{b}}_{ri}\left(k\right)=R_{i}{\vec{b}}_{i}\left(k\right)$$

In order to obtain the orientation and calibration matrix for each sensor *i*, the mean value of the acceleration ${\vec{a}}_{i}\left(k\right)$ (Equation (4)) and magnetic field ${\vec{b}}_{i}\left(k\right)$ (Equation (5)) vectors data are calculated, where *n* corresponds to the number of samples.

(4) $$\bar{{\vec{a}}_{i}}=\frac{\sum_{k=1}^{k=n}{\vec{a}}_{ri}\left(k\right)}{n}$$

(5) $$\bar{{\vec{b}}_{i}}=\frac{\sum_{k=1}^{k=n}{\vec{b}}_{ri}\left(k\right)}{n}$$

To determine the sensor orientation, an orthonormal vector basis was determined for each sensor *i* (***B***_1_), i.e., a set of orthogonal vectors with norm 1. From this basis, the calibration matrix (***M****_i_*) was determined for each of the sensors whose values were used in motion detection. For this purpose, vector 1 is determined in the direction of the average acceleration (Equation (6)), vector 2 is perpendicular to the acceleration and the magnetic field (Equation (7)), and vector 3 is created perpendicular to the previous two vectors (Equation (8)). With this set of vectors, the orthonormal basis of the vectors is obtained (Equation (9)) to finally derive the calibration matrix for each of the sensors (Equation (10)).

(6) $$\vec{v1_{i}}=\frac{\bar{{\vec{a}}_{mi}}}{\left\|\bar{{\vec{a}}_{mi}}\right\|}$$

(7) $$\vec{v2_{i}}=\frac{\vec{v1_{i}}\;\times \bar{\;{\vec{b}}_{mi}}}{\left\|\vec{v1_{i}}\;\times \;\bar{{\vec{b}}_{mi}}\right\|}$$

(8) $$\vec{v3_{i}}=\vec{v1_{i}}\;\times \;\vec{v2_{i}},$$

(9) $$B_{i}=\left\{\;\vec{v1_{i}}\;,\vec{v2_{i}}\;,\vec{v3_{i}}\;\right\},$$

(10) $$M_{i}=B_{i}{}^{-1}B_{2},$$

where i=1, and the matrix for sensor 2 is represented by $M_{2}=I$, due to the fact that the forearm is taken as a reference frame, since sensor 2, located on the ulna and radius (area with less muscle mass) presents less interference and greater reliability, and therefore, allows its alignment with the global axis and serves as a reference to locate sensor 1 (arm), and subsequently others, to allow an analysis of the information of the other body segments of the upper body.

- Step 3: Orientation estimation during movement

In this step, the orientation of the sensor during the movement was estimated by updating the value associated with the accelerations, angular velocities, and magnetic field vectors. In other words, from the information given by the $R_{i}$ and $M_{i}$ rotation matrices, the vectors corresponding to the accelerations (Equation (11)), angular velocities (Equation (12)), and magnetic fields (Equation (13)) were updated.

(11) $${\vec{a}}_{ri}\left(k\right)=M_{i}R_{i}{\vec{a}}_{i}\left(k\right)$$

(12) $${\vec{\omega }}_{ri}\left(k\right)=M_{i}R_{i}{\vec{\omega }}_{i}\left(k\right)$$

(13) $${\vec{b}}_{ri}\left(k\right)=M_{i}R_{i}{\vec{b}}_{i}\left(k\right)$$

The described process is based on the sensor information fusion and is complemented by the adjustment given by a Kalman filter (KF) that is executed in the inertial units to improve the signal quality.

#### 2.2.4. Stage 4. Sensor Assignment and Rotation Settings According to the Analytical Units of Movement

Another important factor related to the signal features generated by the motion capture system is the determination of the location of each sensor used to identify the analytical motion unit and the body segment to which the information corresponds. In this work, as part of the proof-of-concept, two Imocap GIS sensors were used to capture the arm and forearm motion information related to the elbow joint and the pronation-supination motion of the forearm, with each sensor located in a body segment (arm and forearm).

As an example, Table 2 describes the position of the sensors on the right arm and forearm in the initial position, established from the location given by the coordinate system of the sensors.

Considering the information acquired by the sensors (which, depending on the configuration, can be specified in the firmware in the form of Quaternions or Euler angles) and considering the initial position of the sensors on the particular body segment, the inherent rotations are assigned to the type of motion (analytical unit of motion) of the joint or segment of the human body to which they correspond. Considering that each joint of the human body has a different biomechanical model, Table 3 shows the assignment of rotations to the upper extremities of the human body under study. The study was limited to the arm and forearm segments, where the elbow with one degree of freedom can perform the flexion and extension movement, while the forearm can perform the pronation and supination movement.

Table 3 shows that flexion and extension movements of the elbow are performed as a rotation of a delta angle (*δ*) around the anatomical Z axis in the sagittal plane, whose rotation matrix is represented in Table 3. Pronation and supination movements were performed in the transverse plane around the X axis (when the patient was in the upright starting position with arms extended laterally) or around the Y axis (with previous elbow flexion of 90°), and analyzed in the anteroposterior plane. The rotation matrix representing the prone supination movements is also shown (Table 3).

In this way, knowing the position and orientation of each sensor by means of Equations (1)–(13), and with the application of rotation matrices (shown in Table 3), it is possible to determine the measurements of joint amplitude and/or rotation of the body segments.

#### 2.2.5. Stage 5. Model Implementation in a Virtual Environment and Visualization of Kinematic Information

Based on the defined rotation assignment, the model proposed in the previous stage was implemented to visualize the data captured by the Imocap-GIS system in a virtual environment. In this part of the process, two steps were considered: first, the assignment of each sensor to a virtual object that represents a body segment, and second, the management of data reception and motion simulation.

- Step 1: Assignment of each sensor to a virtual body segment and physical sensor location on the test subject.

The defined rotations are associated with the elements or objects of a virtual environment in which the initial (or zero) position for the avatar is determined by the Unity^TM^ video game development engine [55,56] as the “T” position, and from this, the defined calibration body pose is assumed. In order to be able to visualize both the movements performed by the subject (reflected in an avatar) and the corresponding joint amplitude values between body segments, an avatar with human features are chosen to realistically visualize the movements and the proportions of the joint amplitude measurements.

**Figure 5:**
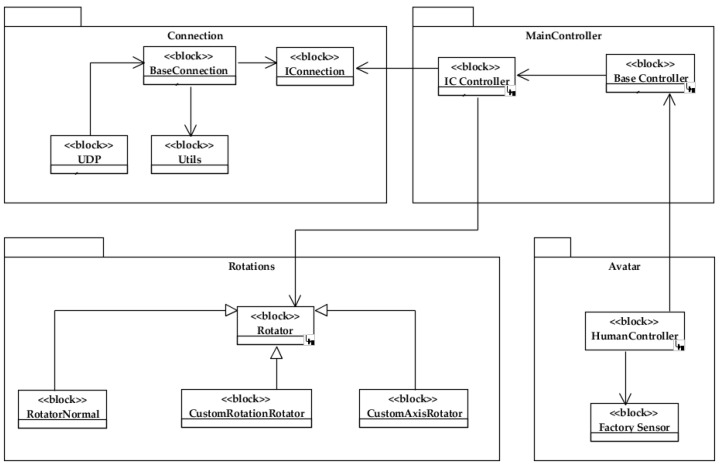
General outline of the visualization model in the virtual environment.

A scheme based on different controllers or software modules (see Figure 5) was implemented to manage the information in the virtual environment. In general, it manages the connections with the sensors of the motion capture system, the body segments to which the sensors can be assigned, and the rotations that each segment can execute.

The purpose of the base controller interface *<<IController>>* is to manage the connection with the sensors, i.e., the process of receiving and then processing the data coming from the motion capture system. Essentially, this controller is responsible for assigning each sensor to a specific body part or segment in the 3D model, as well as assigning the corresponding controller to configure the associated rotations (movements).

The base controller, <<*BaseController*>>, brings together the basic aspects of the data handling and the global usability, especially the initialization of the parameters used in the application of the rotations, as well as the definition of the functions associated with the storage of the motion capture session file.

In the human avatar controller <<*HumanController*>>, the constants that allow for the identification of each body part and the corresponding sensor are created. From this controller, several extensions are derived that allow for the manipulation of the different body segments that can be assigned in the movement management, as well as the axes around which the movement is executed. Additionally, the degrees of rotation, which allow us to obtain the initial position of each sensor (and body part) are controlled.

Finally, the previously defined parameters are established, such as sampling rate, signal reception format (Quaternions or Euler angles), and the signals to be transmitted: accelerometer, gyroscope, and magnetometer values. Subsequently, the assignment and configuration of the sensor are verified physically and visually (comparing the movements of the individual with those of the avatar), and in case of any failure or anomaly, the validation process continues, requiring the pertinent movements to achieve the appropriate calibration. If the validation is correct, data capture proceeds.

- Step 2: Data reception and motion simulation

**Figure 6:**
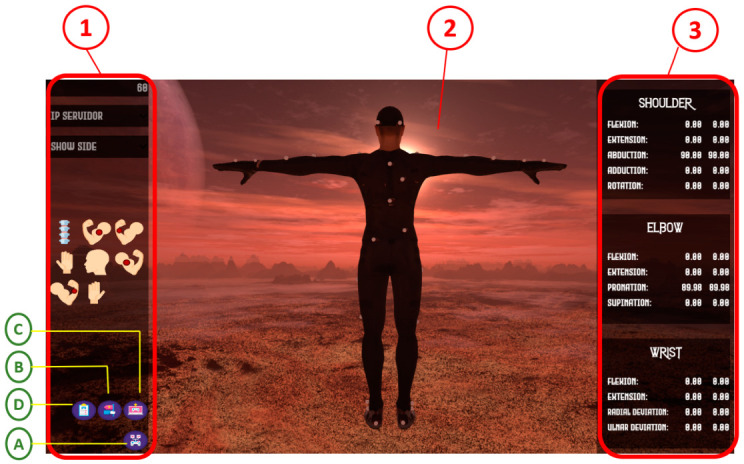
Interface of the virtual environment generated to implement and validate the signal transformation model.

The virtual environment was implemented using the Unity^TM^ video game development engine, based on each of the controllers included in the block diagram in Figure 6. The main components are included in the interface, comprising the components for configuration and visualization of upper-limb kinematic information. As shown in Figure 6, block number (1) includes the configuration and sensor assignment options and information; block number (2) corresponds to the avatar movement visualization area (which simulates the movements of the individual); while block number (3) corresponds to the upper limb kinematic information visualization section.

The sensors start sending data according to the configuration specified in the firmware until the process is stopped or the device battery runs out. The data are stored in a simple text file, where each line is created for each data frame, which is queued as soon as the data are received from the connected sensors. Since the device receives at a rate of 60 frames per second (changeable to 30 or 120), a buffer is used every 120 lines to prevent data loss. This buffer is saved and cleared to resume the data arrangement. The corresponding session is stored, which contains the data associated with the movements of each body segment, and on this basis, the kinematic information of the upper limbs is obtained.

### 2.3. Validation of the Signal Transformation Model

**Figure 7:**
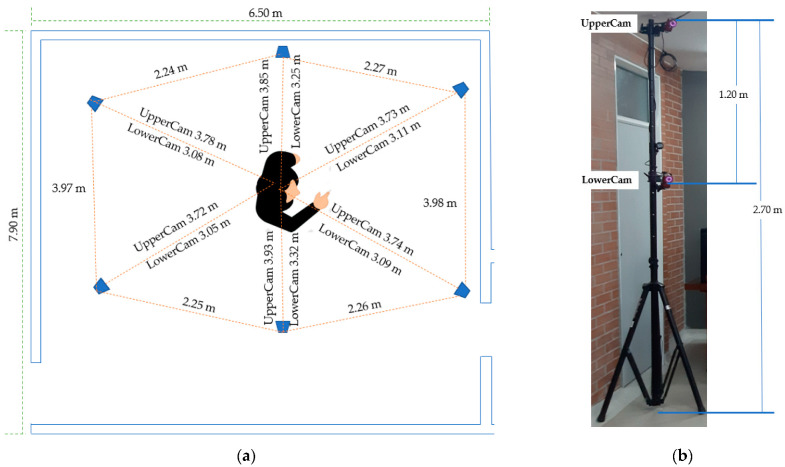
OptiTrack system test scenario: (**a**) optical system camera layout in the test scenario; (**b**) location of the cameras on the tripod.

The proposed method was evaluated in a controlled environment to compare the data associated with joint amplitude and movements of the upper limb. This was carried out using the inertial and magnetic system that implements the proposed signal transformation model, versus an optical system considered as gold standard. The optical system was composed of 12 cameras OptiTrack Flex 13 of 1.3 MP (MegaPixels), and with configurable sampling rates at 30, 60, or 120 FPS (frames per second), distributed (2 for each tripod) as shown in Figure 7, and whose information was accessed visually through the Motive software. For the validation of the model, the following protocol was considered:

- Motion selection: Table 4 shows the protocol for the execution of elbow flexion-extension and forearm pronation-supination movements applied in the experiment, considering the suggestions of professionals in motor recovery therapy.
- Configuration and calibration of the optical motion capture system (OptiTrack/Motive): calibration was carried out in such a way that the cameras detected the reflective markers properly.
- Initialization of the virtual environment and configuration of the inertial and magnetic motion capture system (Imocap-GIS): the virtual environment was initialized to assign the sensors to the body segments (icon A in Figure 6) and based on this assignment, the possible rotations and form of information representation were configured (icon B in Figure 6). It was also possible to define the sampling rate for the session: 30, 60, or 120 samples per second (default 60 Hz, see the upper-left corner of Figure 6).
- Location of the markers and sensors on the body segments: for the optical system, the markers were fixed on the two body segments (rigid body): 3 markers on the arm and 3 markers on the forearm. In the case of the inertial and magnetic system, using elastic bands fixed with hook-and-loop tape, the arm and forearm sensors were placed (Figure 4c), considering the calibration of body posture.
- Initiate recording of session data: for both the optical, inertial and magnetic systems, the process of displaying on-screen information and recording the data associated with the movement of the upper limb was initiated.
- Motion execution and kinematic information visualization: the participant started the execution of movements and, simultaneously, the Motive software screen showed the movement of the body segments formed by the markers. For the inertial and magnetic system software, the movements were simulated by the avatar and the corresponding kinematic information was displayed in a quantitative way (area (3) of Figure 6).
- Session end: at the end of the movement protocol, the recording process was stopped in the two systems, which generated the respective data files.
- Finally, it was necessary to apply the RMSE (root mean square error) equation for the selected motions, and to analyze the results.

## 3. Results

The results of this work focus on two basic elements: first, on the proposal of a model for the conversion of digital signals into kinematic information, and second, on the results of the validation tests of the model. These elements are described below.

### 3.1. Digital Signal to Kinematic Information Transformation Model

**Figure 8:**
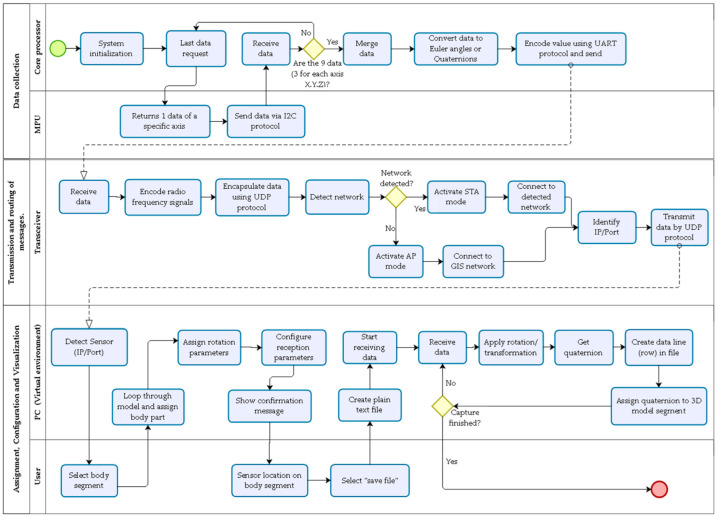
Proposed signal transformation model.

The proposed model summarizes the necessary activities to convert the digital signals generated by the inertial and magnetic motion capture system into kinematic information that can be understood by motor rehabilitation medicine professionals. As shown in Figure 8, the process starts with data acquisition, which mainly involves the sensors and the processing unit, then the transceiver is responsible for data transmission and message forwarding to the PC. Then, the sensors are assigned to the individual body segments where the appropriate configuration is made (sampling rate, signal reception format, and signals to be transmitted). Finally, the simulation of the motion and the quantitative kinematic information of the motion units to be analyzed are obtained visually in the virtual environment.

### 3.2. Results of the Validation Tests of the Proposed Model

The motion execution protocol described in Table 5 was applied, including the initialization and configuration of the optical system and the inertial and magnetic system. The markers and sensors were attached to the patient’s body segments in the center of the test area. Data acquisition and storage took approximately 45.31 s at a sampling rate of 60 Hz. The arm and forearm motion protocol was applied in the sagittal plane for elbow flexion and extension, and the frontal plane for pronation and supination, with elbow flexion at 90° ± 5. The visualization of the movements in real-time and the files with the information related to the behavior of the session were obtained as follows:

- Optical system: a plaintext file was generated, and organized in columns corresponding to the current capture time (first column). Then, for each rigid body, a column associated with the name of the body segment was created, followed by the three columns of the corresponding axes, as follows: Y_Yaw_#rigidbody, X_Pitch_#rigidbody, Z_Roll_#rigidbody (subsequent columns).
- Inertial and magnetic system: a plaintext file containing a column of data for each analytical unit of movement of the upper limb (flexion-extension, pronation-supination). In other words, by applying the transformation model proposed in this work, the value of the joint amplitude or range of motion (ROM) calculated from the value of the positions of the X, Y, and Z axes of each of the sensors were generated.

**Figure 9:**
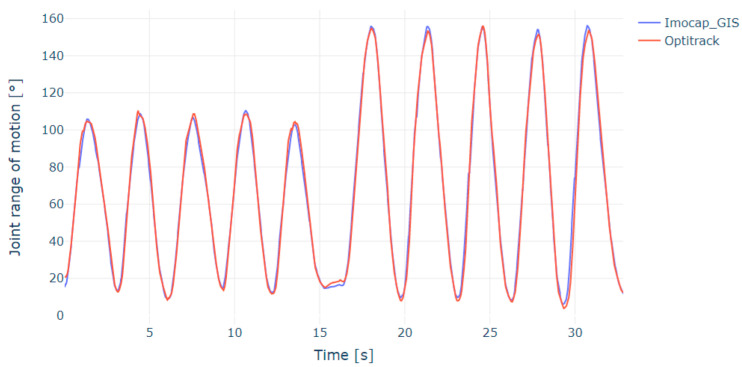
Comparison of OptiTrack vs. Imocap-GIS in flexion-extension motion measured at the elbow joint.

**Figure 10:**
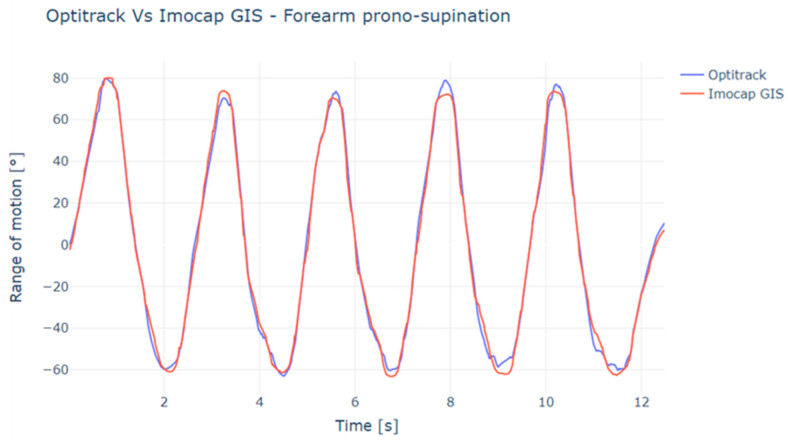
Comparison of OptiTrack vs. Imocap-GIS in prone-supination movement measured on the forearm.

With the captured data, the mean squared error (RMSE) was identified for both the elbow flexion-extension (Figure 9) and forearm pronation-supination (Figure 10) movements, which were obtained with the inertial and magnetic system versus the reference system (optical system).

Applying the equation for the root mean square error calculation, it was found that for elbow flexion and extension, the RMSE was 3.82°, while for prone-supination movements the RMSE was 3.46° (see Table 5). In the same way as the comparison of Imocap-GIS versus OptiTrack, the protocol was applied between OptiTrack and a commercial inertial system, obtaining an RMSE of 2.55° for flexion-extension (1.27° less than with Imocap-GIS) and 4.16° for prone-supination (0.7° more than Imocap-GIS). The above results allowed us to identify that the data obtained with the Imocap-GIS inertial and magnetic system implementing the method of transformation of digital signals to kinematic information offer a high degree of accuracy, usefulness, and an acceptable margin for physical rehabilitation processes.

As expected, the estimated angles are more accurate with the optical motion capture system than with the inertial and magnetic system. Because this optical system uses a set of reflective markers to form a rigid body detectable by the optical system software, it is possible to obtain the triangulation of the position of the body segment in three dimensions, providing higher precision of the angles formed from the center of mass of the rigid body and not from the surface as it is conducted using the inertial systems.

This comparison highlights the challenge of estimating angular kinematics to contribute to motion analysis, and also the challenge of continuing to improve the algorithms to provide greater accuracy and thus greater reliability in making decisions based on the results of motion data capture with the Imocap-GIS system.

It is shown that this estimation is possible with the proposed method, and that the main contribution of the research is to obtain the quantitative values associated with the analytical units of the movement without the need to resort to additional processes, procedures, or calculations.

## 4. Discussion

The usefulness of motion capture systems has gained popularity in different environments, mainly in biomechanics, clinical studies, footwear, 3D animation, sports analysis, rehabilitation, and telerehabilitation [57]. In any of these cases, data processing software or post-processing packages are required to make the generated information understandable in the required context, although specialized knowledge is often needed to achieve this. In this work, a method was presented to transform the digital signals generated by an inertial and magnetic system for motion capture into biomechanical information, specifically associated with the movements of the human upper limb.

Other works have explored this area from other perspectives, such as [19], in which a configuration algorithm was proposed and implemented using a commercial inertial system. In the first part of the algorithm, the calibration phase is performed, requiring a log of stationary motions (a static part and uniaxial motions) to identify at least one pair of unaligned axes in each functional frame of the desired body segments. In this way, reference frames and functional frames for tracking motion about a given axis can be determined. The authors point out the lack of validation tests against a system that is considered a reference standard.

Otherwise, in the model proposed in [21], an inertial system was used for motion capture and the results of the proposed model were validated against an optical system (Vicon) and OpenSim models. The authors presented a model that adapts to gait movements, whereby knee flexion/extension, hip flexion/extension, and hip abduction/adduction were estimated. This work mainly focused on exposing the results of the root mean square error (RMSE), obtained in both the calibration task (in which the lowest error was obtained) and gait task. This method facilitates its application, since it does not require calibration, but it is limited to scenarios where the hip can rotate in all degrees of freedom, and considering the vertical assumption of the pelvic vector, the model is only focused on its application in upright human postures.

On the other hand, the human motion reconstruction method presented in [20] is based on the implementation of two parallel adaptive Kalman filters. For the validation of the experiment, two XSens™ sensors were used to track the human arm motion. It was identified that the orientation method proposed by the authors allows obtaining in real-time the orientation and tracking the movements of the arm and forearm. In this case, the kinematic information is delivered in terms of Euler angles, requiring an additional process to determine the joint amplitude associated with the movements of the upper limb (or the body segment being analyzed).

Another identified aspect is the need to reduce the number of sensors to facilitate data capture in different environments. In [25], a strategy based on two accelerometers located on the forearm is proposed to reconstruct human movement from incomplete data, which allows for obtaining a two-dimensional kinematic model of the arm. In this way, it is possible to obtain information to evaluate the health benefits of monitoring in a residential environment carried out over long periods of time.

On the other hand, ref. [23] describes how lower-limb motion capture can be performed using only one device in the leg, while thigh motions are determined using prediction based on intra-limb coordination neural network. In this work, the orientation matrix was used to determine the acceleration and the movement velocity, and then the position angles (elevation angles) were determined based on the data fusion and using acceleration components. However, rehabilitation processes are not only about reducing the complexity of using motion information acquisition systems, but also about providing other types of information related to the various movements of body segments, facilitating data interpretation tasks to provide timely and accurate information.

Efforts to determine more accurate and readable kinematic information have been presented from different perspectives, and are evidenced in the review presented in [26]. The use of different methods (classified into four categories) used to determine the anatomical axes and to deduce the bony anatomy in the estimation of joint angles is exposed, constituting a significant contribution to the inherent work. It is possible to identify that the proposed method in this work falls into the assumed alignment method category since the IMU sensors of the Imocap-GIS system are located in each body segment. Therefore, the fixed reference frame of the sensor is aligned with the anatomical frame of the body segment to which it is assigned, and from this position the executed movements are determined.

In this sense, and unlike the works presented above, the model for transforming the digital signals generated by the Imocap-GIS motion capture system is divided into the five stages described above. The last stage, related to the graphical presentation of the information of the entire motion capture system, provides considerable assistance to the end-users of the Imocap-GIS system since no specialized knowledge is required to understand the meaning of the displayed quantitative values.

In the validation tests of the proposed transformation model, it can be identified that the digital signals generated by Imocap-GIS provide quantitative information corresponding to the movements of the body segments under analysis. For this specific case, the signals represented correspond to the elbow joint and its flexion and extension movements. Pronation and supination movements associated with the forearm are also included. It should be noted that, with the inherent configurations of the system, the scope can be extended to include the movements of other joints in the human body.

## 5. Conclusions

To establish a model that allows transforming the digital signals generated by a motion capture system into humanly understandable biomechanical information, it is suggested to know and specify the whole process. This implies the understanding from data collection and transmission, through the routing of messages and assignment of rotations according to the body segment from which the data will be captured, until the visualization of kinematic information in an environment easily understood by professionals from different areas without requiring additional technical knowledge.

Several works related to motion science were found in the literature, and their specific contribution to motion capture systems was identified. Most of these works include technical validations with internationally recognized systems, which ensures the validity and reliability of the results obtained. In the case of the model proposed in this work, implemented in the inertial and magnetic system Imocap-GIS, the validation was carried out with the optical system OptiTrack, obtaining an RMSE of 3.6° (averaged between the two movements analyzed). This represents a valid margin of error that should be considered for implementation in motor rehabilitation processes, to contribute and provide information with a high degree of accuracy in a timely and clear manner for the specialists responsible for the motor recovery of patients.

During the validation of the model, it was found that both the Imocap GIS system and the commercial inertial system had a lower RMSE during flexion and extension movements when comparing the behavior during angle measurement with an optical system. In contrast, a higher RMSE resulted for pronation and supination motions. This was due to the fact that flexion and extension were performed around a joint where the axis of motion did not cause major disturbances for data acquisition. During pronation and/or supination movements, not only were the movements of the forearm activated, but the movements of the wrist and hand also involuntarily interfered, often affecting the measurements. Similarly, the accuracy of the data during pronation-supination was affected by the position of the inertial sensors (and the markers in optical systems), since the mobility of the forearm was greater in the area distal to the elbow than in the area proximal to the elbow.

## Figures and Tables

**Table 1:** General Imocap-GIS components.

Item		Device	Description
Hardware	Embedded hardware	Mass Storage	Micro SD card (SDHC card of arbitrary capacity). Can store sensor configurations, calibration data, collected information from sensors, among others.
		Main Processor (SoC)	Interprets firmware instructions by performing basic arithmetic, logic and input/output operations on the system.
		Printed Circuit Board	Performs electrical interconnection of the functional elements of the system through the conductive tracks, and mechanically supports the set of electronic components.
		Power Source and Management	Composed of a LiPo (lithium polymer) battery and an electronic charge regulator.
		Modem and Radio Modules	Provides real-time wireless communication between the motion capture device and a personal computer. It can be implemented with XBEE (ZigBee), Bluetooth, or WiFi modules.
	MPUs	The motion processing units used are Invensense™ MPU-9250 based on MEMS (microelectromechanical systems). This MPU is an SiP technology device with 9 DOF (degrees of freedom) and motion tracking technology (specialized in motion capture) designed for low power consumption, low cost, and high-performance characteristics. Among its main typologies are the combination of a 3-axis gyroscope, 3-axis accelerometer, and 3-axis digital compass (magnetometer) in a single encapsulated chip, together with a DMP (digital motion processor) capable of processing complex onboard data fusion algorithms.	
Firmware	Source code embedded in the hardware that executes the instructions for performing the functions of the motion capture system.		

**Table 2:** Rotation of the axes of each sensor to perform the initial calibration.

Item	Sensor 1: Right Arm		Sensor 2: Right Forearm	
Rotation to place the sensors in the initial position.	1. 270° rotation with a Delta_1_ angle ($\delta_{1}$) around X axis.	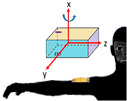 $R\left(\delta \right)=R_{x}\left(\delta_{1}\right)$	1. Rotation with a Delta_3_ angle ($\delta_{3}$) around X axis.	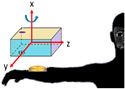 $R\left(\delta \right)=R_{x}\left(\delta_{3}\right)$
	2. Orthogonal rotation with a Delta_2_ angle ($\delta_{2}$) around actual Y axis (y′).	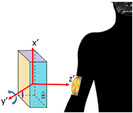 $R\left(\delta \right)=R_{y^{\prime }}\left(\delta_{2}\right)$	2. Orthogonal rotation with a Delta_4_ angle ($\delta_{4}$) around actual Y axis (y′).	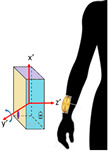 $R\left(\delta \right)=R_{y^{\prime }}\left(\delta_{4}\right)$
Rotation equation	$R\left(\delta \right)=R_{x}\left(\delta_{1}\right)R_{y^{\prime }}\left(\delta_{2}\right)$		$R\left(\delta \right)=R_{x}\left(\delta_{3}\right)R_{y^{\prime }}\left(\delta_{4}\right)$	

**Table 3:** Rotation assignment for flexion-extension and prone-supination motions.

Motion	Definition	Rotation Equation	Rotation Matrix
Flexion- extension.	Rotation in the sagittal plane around the anatomical Z axis (transverse axis).	$R\left(\delta \right)=R_{z}\left(\delta_{n}\right)$	$R\left(z,\;\delta \right)=\left[\begin{array}{ccc} \cos\delta & -\sin\delta & 0\\ \sin\delta & \cos\delta & 0\\ 0 & 0 & 1 \end{array}\right]$ where δ represents the flexion/extension angle.
Pronation- supination.	From the initial position (upright): rotation in the transverse plane around the anatomical X axis (vertical axis).	$R\left(\Phi \right)=R_{x}\left(\Phi_{n}\right)$	$R\left(x,\Phi \right)=\left[\begin{array}{ccc} 1 & 0 & \;\;0\\ 0 & \cos\Phi & -\sin\Phi \\ 0 & \sin\Phi & \cos\Phi \end{array}\right]$ where Φ represents the pronation- supination angle.

**Table 4:** Arm and forearm movements selected for model validation.

Item	Motion	Initial Position	Density/Intensity
1	Flexo-extension	Upper extremities at the sides of the body and palms of the hands facing the body.	- Flexion from ≈0° to ≈90°, and extend to starting position ≈0°: 5 repetitions. - Flexion from ≈0° to the maximum possible and extend to ≈0°: 5 repetitions.
2	Prono- supination	Flex the elbow to ≈90° and position the thumb upward.	Rotate the forearm towards the inside of the midline, i.e., pronate (palm downwards) as far as possible, and then rotate the forearm in the opposite direction so that the palm is facing upwards, i.e., supinated, until the maximum possible value is obtained: 5 repetitions.

**Table 5:** RMSE results between OptiTrack vs. Imocap-GIS.

Motion	RMSE (Degrees)
Elbow flexion-extension	3.82
Forearm pronation-supination	3.46

## Data Availability

Not applicable.
